# Delayed Right Ventricular Automatic Implantable Cardioverter Defibrillator (AICD) Lead Perforation Resulting in Cardiac Tamponade: A Case Study

**DOI:** 10.7759/cureus.68996

**Published:** 2024-09-09

**Authors:** James Hubert, Dylan S Irvine, Imani Thornton

**Affiliations:** 1 Anesthesiology, HCA Florida Westside Hospital, Plantation, USA; 2 Anesthesiology and Critical Care, HCA Florida Westside Hospital, Plantation, USA

**Keywords:** anesthesia considerations, anesthesiology practice, automated implantable cardiac defibrillator (aicd), critical care and hospital medicine, pacemaker lead perforation

## Abstract

Automatic implantable cardioverter defibrillator (AICD) lead perforation is a rare but potentially life-threatening complication. AICD lead perforations are rare, occurring in approximately 0.1%-0.8% of patients, most commonly within 24 hours of the implantation. ICD lead perforations can be acute (within 24 hours of implantation), subacute (between day 1 and day 30), or delayed (>30 days postimplantation). Delayed lead perforations are rare compared to acute and subacute lead perforations and are not as well-studied because patients are often asymptomatic and are not diagnosed. Here, we report the case of a 44-year-old male who presented to the emergency department with pleuritic chest pain and dyspnea one-month status-post dual-chamber AICD. The patient demonstrated signs and symptoms of cardiac tamponade, which was confirmed with a 2D echocardiogram and computed tomography (CT) scans. Emergency pericardiocentesis was performed under general anesthesia, which restored hemodynamic stability. The right ventricular lead was repositioned and a pericardial drain was placed. The patient remained in the intensive care unit (ICU) for three days and was discharged to home on postoperative day 8.

## Introduction

Cardiac perforation by the leads of cardiac implantable electronic devices (IEDs), such as automatic implantable cardioverter defibrillators (AICDs), is a rare but potentially critical complication associated with the implantation of these devices [1-4]. Cardiac perforation by cardiac IEDs occurs in 0.1%-0.8% of patients, most commonly within 24 hours of implantation [4]. When this complication occurs, it is characterized as acute (within 24 hours of implantation), subacute (24 hours-30 days of implantation), or delayed (>30 days following implantation) [1-4]. Lead perforations are recognized mostly during the acute phase [2]. Subacute and delayed perforations are less common [2]. We report a case of delayed cardiac perforation of the right ventricular wall one month after dual-chamber AICD placement. Risk factors that have been associated with increased risk of lead perforations include female sex, steroid use, advanced age, and low body weight [4]. The pathophysiology of cardiac perforation by cardiac IEDs depends on the time since implantation. Most lead perforations occur during the acute phase at the time of implantation due to the lead perforating into the pericardium [4-5]. This most commonly occurs in the right ventricular apex due to the presence of a thin wall [4-5]. Delayed subacute and delayed perforations are more rare and have been theorized to occur due to mechanisms such as slow lead advancement during cardiac contractions resulting in dissection of cardiac muscle layers or due to tension at the lead tip that prevents movement in conjunction with contractions of the heart, causing erosion and perforation [4,6-7]. The management of cardiac perforation by cardiac IED leads may include open surgical intervention, transvenous lead extraction, lead adjustment and re-implantation, or conservative management [2,4,8]. The approach to management often depends on patient status such as symptomatology, the presence of hemodynamic instability, or the presence of complications such as pericardial effusion [8]. 

## Case presentation

We report the case of a 44-year-old male who presented to the emergency department one-month status-post dual-chamber AICD implantation with a chief complaint of pleuritic chest pain and dyspnea. The patient had a past medical history of coronary artery disease (CAD) and ischemic cardiomyopathy with an ejection fraction (EF) of 30%-35% as well as hyperlipidemia and hypertension. Patient home medications included atorvastatin, buspirone, clopidogrel, rivaroxaban, sotalol, spironolactone, and sacubitril/valsartan. The patient had an allergy to penicillin. The patient’s surgical history included vasectomy, lithotripsy, and ICD placement, all without anesthetic complications. The patient is a lifetime nonsmoker. At the time of presentation to the emergency department, patient vitals included a heart rate (HR) of 71 beats per minute (bpm), respiratory rate of 20 breaths per minute, blood pressure (BP) of 88/53 millimeters mercury (mmHg) with a mean arterial pressure (MAP) of 64, and oxygen saturation (SpO2) of 97%. Upon physical exam, it was noted that the patient had a physiology that was concerning for cardiac tamponade, including tachycardia, hypotension, jugular venous distension (JVD), and muffled heart sounds. The patient was alert and oriented to person, place, and time, and the rest of the physical examination was within normal limits. 

**Figure 1:**
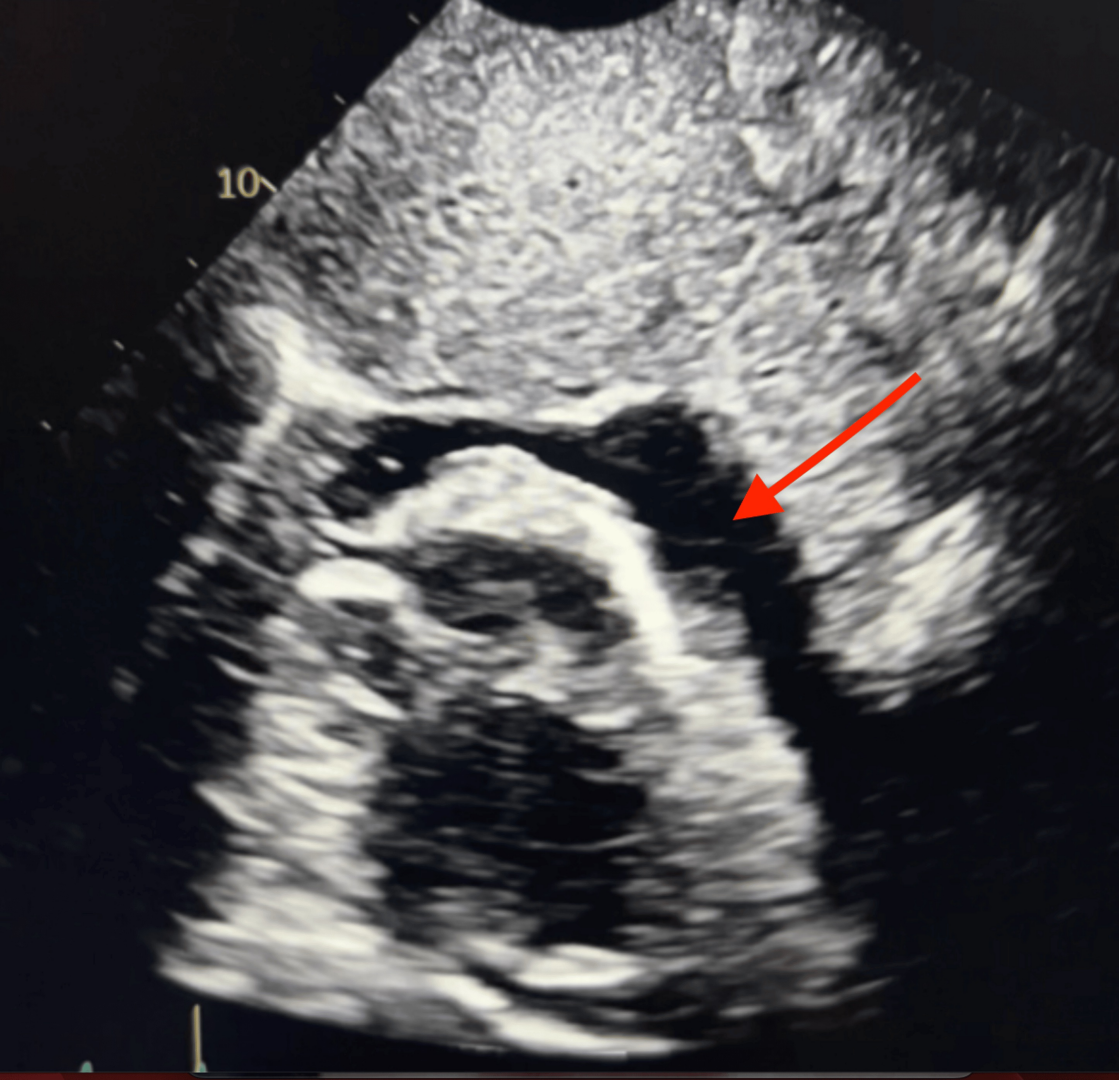
Two-dimensional (2D) echocardiography obtained in the emergency department (ED), demonstrating large pericardial effusion

**Figure 2:**
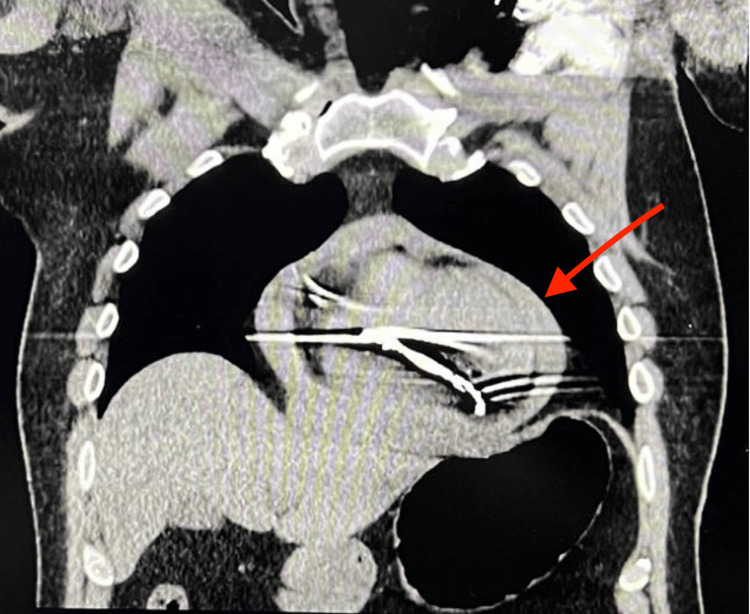
Computed tomography (CT) imaging obtained following the patient being admitted to the emergency department, confirming large pericardial effusion

A two-dimensional (2D) echocardiography demonstrated a large pericardial effusion, which is demonstrated in Figure 1. This diagnosis was also confirmed with computed tomography (CT) of the chest, which also showed a large pericardial effusion, shown in Figure 2.

An electrophysiologist was consulted, and the patient was scheduled for an emergency pericardiocentesis to be performed under general anesthesia. Preoperative electrocardiogram completed in the emergency department showed normal sinus rhythm. Airway examination was within normal limits. Anesthesia was induced with ketamine 60 milligrams (mg) and etomidate 20 mg in an effort to preserve cardiac function. Rapid sequence induction (RSI) was performed using succinylcholine 120 mg as the patient was not NPO prior to the induction of anesthesia. The patient was mechanically ventilated using low tidal volumes of 450 milliliters (ml), without the addition of positive end expiratory pressure (PEEP) to prevent reduced venous return.

Immediately prior to evacuation via emergency pericardiocentesis, the patient's vitals were a BP of 105/60 mmHg, an HR of 121 bpm, and an SpO2 of 100%. Emergency pericardiocentesis evacuated 700 mL of blood. Immediately following pericardiocentesis, the patient’s hemodynamic instability improved, and vitals were a BP of 110/55 mmHg, an HR of 64 bpm, and an SpO2 of 100%. The right ventricular lead was repositioned, and a pericardial drain was placed. 

**Figure 3:**
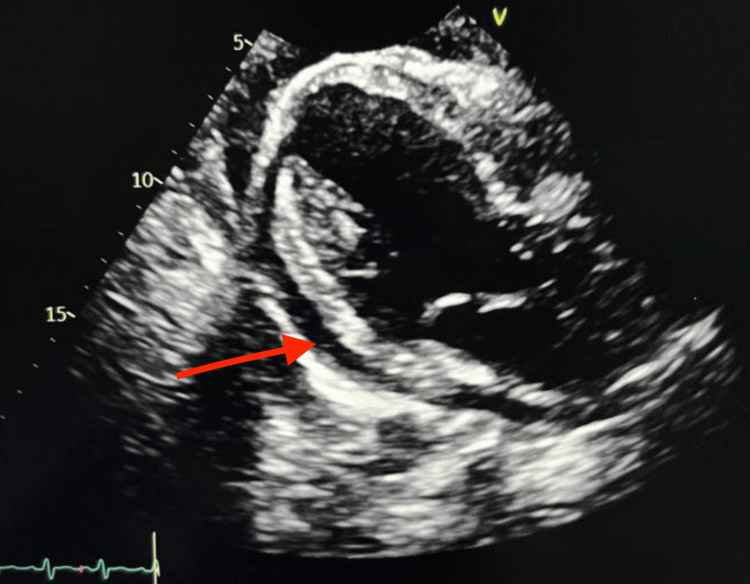
Follow-up echocardiogram taken postoperatively, which demonstrated an ejection fraction (EF) of 20%-25% with trace pericardial effusion and associated hematoma

Follow-up echocardiogram showed an EF of 20%-25% with trace pericardial effusion and associated hematoma, which is demonstrated in Figure 3. The patient remained stable in the intensive care unit for three days postoperatively and was subsequently discharged home on postoperative day 8.

## Discussion

Myocardial perforation following placement of IEDs such as AICDs is a rare complication, with an estimated incidence in 0.1%-0.8% of patients [4]. Although rare, myocardial perforation following IED placement can result in serious complications in patients, such as rapid hemodynamic instability, chest pain and dyspnea, and pericardial effusion [2,4,8]. These complications often result in emergent surgical intervention and require specific anesthetic and surgical considerations to restore hemodynamic stability and optimize patient outcomes. AICD lead perforations most commonly occur within 24 hours of the implantation and are characterized as acute (within 24 hours of implantation), subacute (between day 1 and day 30), or delayed (>30 days postimplantation) [1,4,9]. The diagnosis of myocardial perforation following placement of IEDs is clinical and may include signs of pericardial effusion, cardiac tamponade, hemodynamic instability, dyspnea, or chest pain in the setting of a patient history of recent or remote IED placement [1-4].

Most myocardial perforations occur at the time of device implantation with the lead exiting the right ventricle into the pericardium [1,9]. The right ventricular apex has been reported the most common site of perforation due to the thin wall [3,10]. Delayed perforation, however, is quite rare and is defined as perforation that occurs after more than one month [1,4]. Postimplantation pericardial effusion can be caused by a traumatic inflammation of the myocardium or irritation of the visceral pericardium via immune‐mediated mechanisms, but it can result from a lead perforation, especially in the presence of risk factors such as age, female sex, and concomitant therapies such as anticoagulant or antiplatelet medication [3]. Other mechanisms that have been proposed to result in delayed lead perforation include slow lead advancement during cardiac contractions resulting in dissection of cardiac muscle layers or due to tension at the lead tip that prevents movement in conjunction with contractions of the heart, causing erosion and perforation [4,6-7]. Our patient developed right ventricular lead perforation and subsequent cardiac tamponade, which was exacerbated by the patient’s prescribed anticoagulant medications clopidogrel and rivaroxaban.

The management question of late lead perforation is whether to extract the lead or not. If it is decided to extract or reposition the lead responsible for the perforation, this can be done through open surgical intervention, percutaneous intervention, or through transvenous adjustment. In select cases, conservative management is utilized, but this is not common [2,5,11]. The approach to management often depends on patient status such as symptomatology, the presence of hemodynamic instability, or the presence of complications such as pericardial effusion [2,5]. Surgical intervention is the treatment of choice in the case of hemodynamic instability, rapid progression of pericardial effusion, or if closed pericardiocentesis fails [2,11]. As for our case, the patient had a large, progressive effusion causing cardiac tamponade with severe chest pain and dyspnea. This case was successfully managed with closed pericardiocentesis, a pericardial drain was placed, and repositioning of the offending lead. The cardiothoracic team was on standby for emergency backup thoracotomy if required. Fortunately, this was not needed in our case.

In cases of cardiac perforation by IED leads, special considerations must be made for the intraoperative administration of anesthesia. The anesthetic goals in such cases are to maintain spontaneous ventilation and keep the heart full (maintain preload), fast (maintain HR), and tight (maintain SVR and BP) [12-13]. We chose to use ketamine as our primary induction agent. By utilizing ketamine for induction, we allowed the maintenance of HR, contractility, and SVR and encouraged spontaneous ventilation. We chose to supplement ketamine with etomidate for induction, because etomidate has minimal cardiac depression and respiratory effects. The ventilation strategy was to use low tidal volumes without PEEP as positive pressure ventilation may decrease venous return, resulting in decreased stroke volume and cardiac output. After the tamponade was evacuated, we were prepared for rebound hypertension with vasodilators and beta-blockers and were prepared to rapidly deepen the depth of anesthesia if needed, but patient hemodynamics were stable, and a resolution of tachycardia was observed without rebound hypertension.

This case highlights the importance of a high index of suspicion for cardiac perforation and cardiac tamponade in patients with chest pain, dyspnea, hemodynamic instability, and signs of pericardial effusion in the setting of a history of recent or remote cardiac IED placement. Rapid recognition and treatment of this complication can improve outcomes and prevent morbidity and mortality in these patients. Special anesthetic considerations should be made when considering the anesthesia care plan to limit cardiac suppression and maintain cardiac preload, HR, BP, and SVR. Further research is needed to study delayed cases of cardiac perforation by IED leads, to guide treatment protocols and optimize patient outcomes.

## Conclusions

This case report describes the presentation of delayed right ventricular perforation by AICD leads complicated by a cardiac tamponade in a 44-year-old male patient. The patient was managed surgically with emergent pericardiocentesis followed by the placement of a pericardial drain as well as with repositioning of the AICD lead. Postoperatively, the patient was managed in the ICU prior to being discharged home. This report highlights the importance of a more cautious approach in a patient with a history of implanted cardiac leads presenting with chest pain or dyspnea, to prevent overlooking cardiac lead perforations. According to our case, this fatal complication can be successfully managed by closed pericardiocentesis and RV lead repositioning, but careful hemodynamic and echocardiographic monitoring is necessary because of the risk of delayed re-tamponade as the site of perforation may not be fully closed. Further, tailoring the intraoperative anesthesia plan can improve outcomes for patients by promoting cardiac and hemodynamic stability intraoperatively.
